# Differential regulation of long noncoding RNAs by endogenous and exogenous reactive oxygen species-generating prooxidants in NIH3T3 cells

**DOI:** 10.1371/journal.pone.0333072

**Published:** 2025-09-22

**Authors:** Yoichi Kurokawa, Takashi Honda, Shingo Fujii, Yugo Narisawa, Hirotaka Ogawa, Hiroki Kawashima, Hidenori Tani

**Affiliations:** 1 Department of Bioscience and Biotechnology, Fukui Prefectural University, Eiheiji-cho, Fukui, Japan; 2 Department of Gastroenterology and Hepatology, Nagoya University Graduate School of Medicine, Showa-ku, Nagoya, Japan; 3 Department of Health Pharmacy, Yokohama University of Pharmacy, Yokohama, Japan; 4 Nagoya Industrial Science Research Institute, Nagoya, Aichi, Japan; Fujita Health University: Fujita Ika Daigaku, JAPAN

## Abstract

Long noncoding RNAs (lncRNAs) play crucial roles in various cellular processes, including the response to oxidative stress. However, the relationship between oxidants and lncRNA expression remains poorly understood. This study investigated the effects of endogenous and exogenous prooxidants with reactive oxygen species (ROS)-generating activity on lncRNA expression in NIH3T3 cells. We treated cells with various compounds, including food-derived polyphenols and environmental pollutants, for 24 hours and analyzed lncRNA expression. Treatment with 1 μM exogenous prooxidants (1,2,4-benzenetriol or 1,4-naphthoquinone) resulted in a significant increase (>50-fold) in the expression of several lncRNAs, including Snhg4, Kcnq1ot1, Rmst, Neat1, Gt(ROSA)26Sor, and Peg13. Conversely, exposure to endogenous prooxidants (dopamine and adrenaline), certain food-derived polyphenols (chrysin and 3-*O*-methylquercetin), or 1,2-naphthoquinone led to a significant decrease (<0.5-fold) in Rmst expression. Similarly, Neat1 expression was significantly reduced in the presence of food-derived polyphenols (luteolin, quercetin, and piceatannol). These findings suggest that prooxidants with distinct redox properties differentially regulate lncRNAs and potentially influence oxidative stress responses. Our results provide new insights into the complex interplay between oxidants and lncRNA regulation. This may have implications for understanding oxidative stress-related pathologies and developing novel therapeutic strategies.

## Introduction

Long noncoding RNAs (lncRNAs) are a large and diverse set of transcribed RNA molecules that are more than 200 nucleotides long and do not possess complete open reading frames. However, recent studies have unveiled the abundant transcription of lncRNAs within cells and their dynamic regulation of intracellular gene expression mechanisms. These lncRNAs have been found to be involved in nuclear structures, chromatin remodeling, imprinting, transcription, translation, and epigenetic control [1–3]. However, the functions of lncRNAs have been only partially elucidated, and much remains to be examined regarding the thousands of lncRNAs with uncharacterized functions.

Pioneer studies conducted by two independent research groups, including the authors, revealed important findings about lncRNAs [4,5]. Short-lived lncRNAs (half-life less than 4 h) primarily consist of well-characterized regulatory lncRNAs. In contrast, long-lived lncRNAs (half-life exceeding 4 h) predominantly comprise housekeeping-like RNAs. These findings suggest that the temporal stability of lncRNAs is related to their functional significance.

We have recently identified novel short-lived lncRNAs in mouse livers that exhibit altered expression in metabolic associated steatohepatitis (MASH) induced by a western diet (WD) [6]. Two of these lncRNAs, Kcnq1ot1 and Rmst, showed dramatic expression changes not only in WD-induced MASH livers but also in CCl_4_-induced MASH livers and fibrotic livers. These lncRNAs may serve as potential markers for hepatic diseases, which are often associated with oxidative stress.

Recent studies have demonstrated that oxidative stress plays a crucial role in modulating lncRNA expression and function [7]. Oxidative stress, characterized by an imbalance between the production of reactive oxygen species (ROS) and the cellular antioxidant defense mechanisms, has been implicated in various pathological conditions, including cancer, neurodegenerative diseases, and cardiovascular disorders [8].The interplay between oxidative stress and lncRNAs has emerged as a key area of research, with growing evidence suggesting that lncRNAs can act as both regulators and targets of oxidative stress responses.

Several lncRNAs have been identified as important players in the cellular response to oxidative stress. For instance, we have demonstrated that certain short-lived lncRNAs, including OIP5-AS1, FLJ46906, LINC01137, and GABPB1-AS1, showed significantly upregulated expression following exposure to hydrogen peroxide (oxidative stress) in human HepG2 cells [9]. These lncRNAs may serve as useful indicators of chemical stress responses, with their levels increasing due to stress-induced prolongation of their decay. In another study, we identified lncRNAs such as GABPB1-AS1 and LINC00152 that responded rapidly to oxidative stress induced by hydrogen peroxide in human induced pluripotent stem cells, suggesting their potential as surrogate indicators for chemical stress responses [10].

Epicatechin gallate [11,12], resveratrol [13] or quercetin [14], phytochemicals, are known to downregulate the expression of lncRNAs. These compounds are reported as prooxidants that can generate ROS under certain conditions and exhibit potential carcinogenicity [15]. These findings highlight the potential of lncRNAs as therapeutic targets or biomarkers in oxidative stress-related diseases.

**Fig 1:**
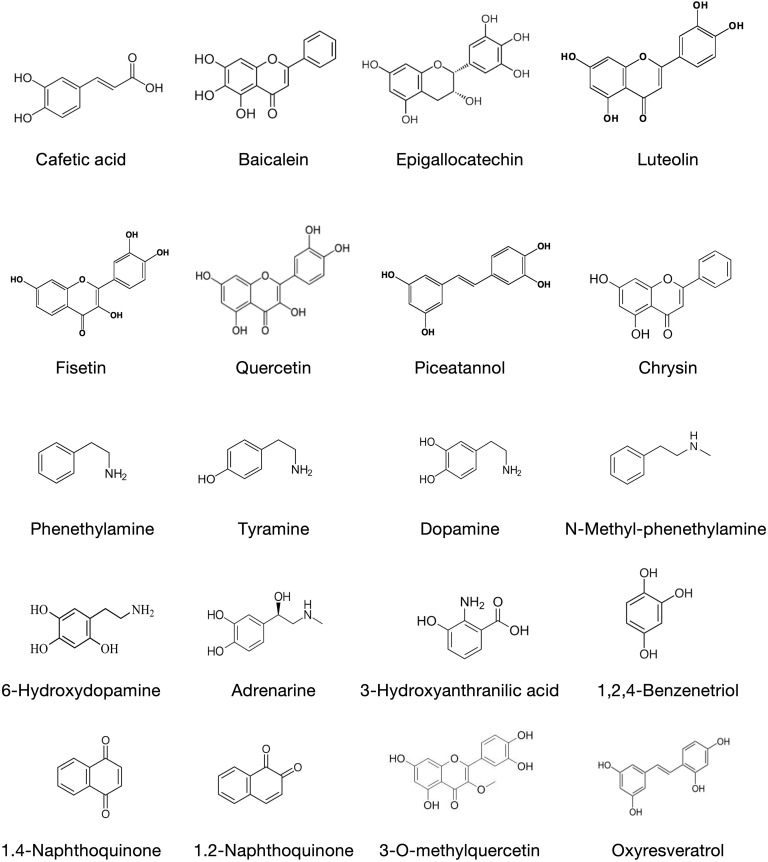
List of the chemical structures of the compounds used in this study.

Despite these advances, the precise mechanisms by which oxidative stress influences lncRNA expression and how different oxidants may differentially modulate lncRNA levels remain poorly understood. Furthermore, the potential role of food-derived compounds and environmental pollutants in modulating lncRNA expression through oxidative stress pathways has not been extensively explored. Such compounds possess prooxidant activity that can generate ROS in the presence of transition metal salts and can cause oxidative stress [16,17]. We selected 20 prooxidant compounds with different structures (Fig 1). Phenylpropanoids [17], flavonoids [18] such as flavones, flavanols, and flavan-3-ols, and stilbenoids [19] are plant-derived antioxidants with prooxidant activity. Aromatic amines [20–22] and 3-hydroxyanthranilic acid [23] are aromatic amino acid-derived metabolites with prooxidant activity. 1,2,4-benzenetriol (hydroxyhydroquinone), produced during the roasting process of coffee beans, can generate ROS [24]. 1,2-naphthoquinine and 1,4-naphthoquinone are exogenous pollutants contained in ambient particulate matter (PM) that generate ROS and can cause adverse health effects in humans [25]. Some flavonoids are known to exhibit both antioxidant and prooxidant activities [18,19]. Understanding the relationships between prooxidant compounds and lncRNAs could provide valuable insights into the molecular mechanisms underlying the health effects of various dietary components and environmental exposures.

In this study, we aimed to investigate the effects of both endogenous and exogenous prooxidants with ROS-generating activity on lncRNA expression in NIH3T3 cells. NIH3T3 cells are a commonly used mouse fibroblast cell line, known for their ability to be easily cultured and their sensitivity to transformation by oncogenes. We selected these 10 lncRNAs, including Kcnq1ot1 and Rmst, based on our previous findings that these lncRNAs increase in CCl4-induced mouse MASH livers and fibrotic livers, which establishes their relevance to oxidative stress responses [6].

By examining a range of compounds including food-derived polyphenols and environmental pollutants, we sought to elucidate the complex relationships between oxidative stress, lncRNA regulation, and cellular responses. Our findings will contribute to a better understanding of the role of lncRNAs in oxidative stress-related pathologies and potentially inform the development of novel therapeutic strategies targeting these regulatory RNA molecules.

## Materials and methods

### Chemicals

1,2,4-Benzenetriol (hydroxyhydroquinone), caffeic acid, dopamine, epigallocatechin, 6-hydroxydopamine, and luteolin were purchased from FUJIFILM Wako Pure Chemical Corporation.

Fisetin, 3-hydroxyanthranilic acid, *N*-methylphenethylamine, 1,2-naphthoquinone, 1,4-naphthoquinone, phenethylamine, tyramine, piceatannol, and oxyresveratrol were purchased from Tokyo Chemical Industry, Co., Ltd. Adrenaline (epinephrine), baicalein, 3-*O*-methylquercetin, and quercetin were purchased from Sigma-Aldrich. Chrysin was purchased from Indofine Chemical Company. These chemicals were dissolved in dimethyl sulfoxide (DMSO; FUJIFILM Wako Pure Chemical Corporation) and diluted in culture medium at a final concentration of 1 μM.

### Cell culture

NIH3T3 cells were cultured in Dulbecco’s modified Eagle’s medium (DMEM) (FUJIFILM Wako Pure Chemical Corporation) supplemented with 10% fetal bovine serum (FBS) and antibiotics at 37 °C in a humidified incubator with 5% CO_2_.

### RNA isolation

Total RNA was extracted from cells using TRIzol Reagent (Thermo Fisher Scientific), according to the manufacturer’s instructions.

### Reverse transcription – quantitative real-time polymerase chain reaction (RT-qPCR)

The isolated RNA was reverse transcribed into cDNA using PrimeScript RT Master Mix (Perfect Real Time) (TaKaRa Bio Inc., Japan). The resulting cDNA was amplified using the primer sets listed in Table 1, and the levels were normalized relative to glyceraldehyde-3-phosphate dehydrogenase (GAPDH) and β-actin (ACTB) mRNAs. The primer specificity was verified using Primer3Plus with BLAST analysis to ensure that our primer sets are designed to amplify conserved fragments that capture all isoforms of each target lncRNA. Relative RNA quantities were calculated as treated values normalized relative to untreated values. We have normalized the changes in ACTB based on GAPDH, that is, normalized so that the relative quantity becomes 1. THUNDERBIRD SYBR qPCR mix (Toyobo, Japan) was used in accordance with the manufacturer’s instructions. For significance testing, the Student’s t-test was used. RT-qPCR analysis was performed using a Quantstudio 7 Flex (Thermo Fisher Scientific). For RT-qPCR, the sample volume was 10 μL, and three independent replicates were performed.

**Table 1:** Primer pairs for RT-qPCR.

Name	Sence sequence (5’-3’)	Antisence sequence (5’-3’)
Gapdh	TGGTGAAGCAGGCATCTGAG	TGAAGTCGCAGGAGACAACC
Actb	CCAGCCATGTACGTAGCCAT	CGGAGTCCATCACAATGCCT
Snhg4	GCATTTTCAGAGTCAACATTCCCA	GAAGCCTCAAGACCTAAACTCCA
Kcnq1ot1	GGCGCAGGTCTGTAATCGTA	CCCAGCAAGGTCCTGAACAT
Rmst	GCAGCTAAAGGACAGGAGCA	TTAACTCGCAGTTGGTGGCA
Neat1	AGTGTGAGTCGTAGCAGTGC	TCACTGTGTAGGCGTCAACC
Gas5	ATGGTGGAGTTTGAGGCTGG	CAAGCAAGCCAGCCAAATGA
Mir17hg	GCACTTGTTCAGTTCCGCAC	CCACAGCTGTTTTGCCAACA
Gt(ROSA)26Sor	GCCACACATGTCCCATTCCA	AACTTTCCAACCCACCACCC
Snhg8	CACAAGGTGGCTATGGTGCT	TCGTCGCGCTAACCTTCAC
Snhg17	TGTCTCCCAAGAGCTCCAGT	ATCCTGCTATGGCCTCCTGA
Peg13	GATTCCACTTGGGTGCCTCA	GACGTCTGCCTGGTCTCTTC

## Results

The expression levels of lncRNAs were differentially induced in the presence of prooxidants.

A previous study recently identified ten lncRNAs with short half-lives t_1/2_ ≤ 4h (Snhg4, Kcnq1ot1, Rmst, Neat1, Gas5, Mir17hg, Gt(ROSA)26Sor, Snhg8, Snhg17, Peg13) in the mouse Neuro-2a cell line [4] (Table 1). Here, we examined whether the expression of these ten lncRNAs was induced when NIH3T3 cells were treated with 1 μM of 20 prooxidants.

**Fig 2:**
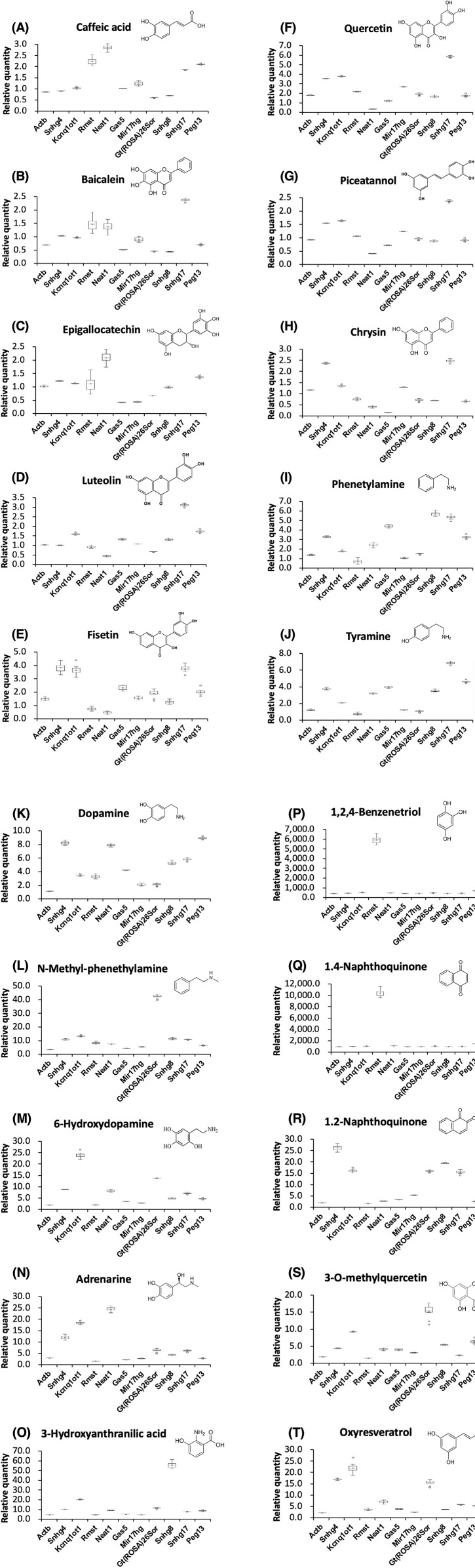
NIH3T3 cells were treated with 20 chemicals for 24 h. Expression levels of the indicated RNAs were determined by RT-qPCR. GAPDH and ACTB were used for normalization. Values represent the mean ± SD obtained from three independent experiments (**P < 0.01, Student’s t-test). The Y-axis indicated that the expression levels of treated cells were divided by those of untreated cells. Thus, one indicated that there was no change in expression levels comparing the untreated cells.

When cells were treated with caffeic acid, a phenylpropanoid, the expression levels of four lncRNAs (Rmst, Neat1, Snhg17, and Peg13) increased by more than 1.5-fold, whereas that of Gt (ROSA)26Sor decreased by 0.5-fold (Fig 2A). Epigallocatechin, a catechin, decreased the expression level of three lncRNAs (Gas5, Mir17hg, and Gt(ROSA)26Sor) to less than 0.5-fold but increased those of two lncRNAs (Neat1 and Snhg17) by more than 1.5-fold (Fig 2C). Among flavonoids, flavones (baicalein, luteolin, and chrysin) increased the expression level of Snhg17 by more than 2-fold (Fig 2B,2D,2H); flavonols (fisetin, quercetin, and 3-*O*-methylquercetin) increased the expression level of five lncRNAs (Snhg4, Kcnq1ot1, Mir17hg, Gt(ROSA)26Sor, and Peg13) by more than 1.5-fold (Fig 2E,2F,2S). Among stilbenoids, piceatannol increased the expression level of Snhg4 by more than 1.5-fold; oxyresveratrol increased the expression levels of six lncRNAs (Rmst, Neat1, Gas5, Snhg8, Snhg17, and Peg13) by approximately 3- to 8-fold and three additional lncRNAs (Snhg4, Kcnq1ot1, and Gt(ROSA)26Sor) by approximately 18- to 28-fold (Fig 2G, 2T). Among aromatic amines, phenethylamine, tyramine, and dopamine increased the expression levels of Kcnq1ot1 or Gt(ROSA)26Sor by approximately 1.4-2.9-fold or 1.2-2.1-fold respectively (Fig 2I, 2J, 2K), whereas *N*-methyl-phenethylamine, 6-hydroxydopamine, and adrenaline increased those of Kcnq1ot1or Gt(ROSA)26Sor by approximately 11.9-26.3-fold or 5−45 fold respectively (Fig 2L, 2M, 2N). Furthermore, 3-hydroxyanthranilic acid, a tryptophan metabolite [23], increased the expression levels of five lncRNAs (Snhg4, Neat1, Gas5, Gt(ROSA)26Sor, and Snhg17) by approximately 1.8-9.6-fold; it also significantly increased those of three other lncRNAs (Kcnq1ot1, Peg13, and Snhg8) by approximately 20–64-fold (Fig 2O). Among the twenty chemicals examined in this study, quinones most strongly induced various lncRNAs; specifically, 1,2,4-benzenetriol [24], as well as 1,2-naphthoquinone and 1,4-naphthoquinone [25], increased the expression levels of seven lncRNAs (Snhg4, Kcnq1ot1, Gas5, Mir17hg, Gt(ROSA)26Sor, Snhg8, and Snhg17) by more than 2-fold each (Fig 2P, 2Q, 2R). Most strikingly, 1,2,4-benzenetriol and 1,4-naphthoquinone increased the expression levels of Rmst, Neat1, and Peg13 approximately 69−10,000-fold, whereas 1,2-naphthoquinone only marginally increased the expression level of these lncRNAs Table 2.

**Table 2:** The 10 short-lived lncRNAs that were investigate in this study.

Name	Accession No.	Length (nt)	*t*_1/2_*
Snhg4	NR_038073	1,050	0.32
Kcnq1ot1	NR_001461	90,612	1.60
Rmst	NR_028262	2,687	1.38
Neat1	NR_003513	3,190	0.01
Gas5	NR_153812	501	0.18
Mir17hg	NR_029382	2,339	0.07
Gt(ROSA)26Sor	NR_027010	613	0.12
Snhg8	NR_028574	785	0.14
Snhg17	NR_015463	3,175	0.48
Peg13	NR_002864	4,723	0.67

*These values are taken from a previous report^4^.

### The expression levels of two lncRNAs — Kcnq1ot1 and Rmst — were differentially affected by prooxidants

We recently reported two lncRNAs (Kcnq1ot1 and Rmst), whose expression levels significantly decrease in mouse models of chronic liver diseases [6]. In this study we investigated how these two lncRNA differ upon treatment with twenty prooxidants in mouse NIH 3T3 cells. Five compounds —luteolin, fisetin, quercetin, phenethylamine, and tyramine— increased Kcnq1ot1 expression level more than approximately 1.5-5-fold (Fig 2D, 2E, 2F, 2I and 2J), nine compounds —*N*-methyl phenethylamine, 6-hydroxydopamine, adrenaline, 3-hydroxyanthranilic acid, 1,2,4-benzenetriol, 1,4-naphthoquinone, 1,2-naphthoquinone, 3-*O*-methylquercetin, and oxyresveratrol — significantly elevated Kcnq1ot1 abundance ranging from about 10-fold to nearly 100-fold increase; none decreased its abundance below the baseline level (Fig. 2L, 2M, 2N, 2O, 2P, 2Q, 2R, 2S, and 2T). Six compounds —caffeic acid, baicalein, quercetin, tyramine, *N*-methyl phenethylamine, and oxyresveratrol— increased Rmst expression level more than approximately 1.5-3-fold, two compounds (1,2,4-benzenetriol, and 1,4-naphthoquinone) increased the expression level more than approximately 6,600−10,400-fold (Fig 2P and 2Q), whereas five compounds (chrysin, dopamine, adrenaline, 1,2-naphthoquinone, and 3-*O*-methylquercetin) decreased the expression level of the lncRNA by less than 0.5-fold (Fig 2H, 2K, 2N, 2R and 2S).

### Quinones — 1,2,4-benzenetriol and 1,4-naphthoquinone — were strong-inducers of lncRNA expression

When comparing two naphthoquinones regarding their effects on inducing lncRNA expressions, both compounds commonly elevated seven out of ten lncRNAs at comparable magnitudes (Fig 2Q and 2R). However, notably only one compound, namely 1,4-naphthoquinone demonstrated robust induction across all ten analyzed lncRNAs, whereas 1,2-naphthoquinone induced only seven lncRNAs at comparable magnitudes (Fig 2Q and 2R). Additionally, among all twenty tested prooxidants, 1,2,4-benzenetriol, was also identified as another strong-inducer capable of elevating all ten lncRNAs examined above 3-fold, indicating broad spectrum activity across multiple targets (Fig 2P).

## Discussion

In the present study, we examined how endogenous or exogenous prooxidants (food-derived compounds or environmental pollutants) modulate the expression levels of lncRNAs in mouse NIH3T3 cell line, to estimate the possible roles of prooxidants *in vitro* through oxidative stress responses. Our research revealed that prooxidants with different redox properties significantly increased the expression levels of lncRNAs *in vitro*, suggesting a possible role of oxidative stress in inducing the expression of lncRNAs and highlighting the importance of identifying potential “stressors” for altered expression levels of lncRNAs. Notably, a by-product of coffee bean roasting and an environmental pollutant (1,2,4-benzenetriol and 1,4-naphthoquinone) with ROS-generating activity were found to significantly increase the expression levels of at least seven out of ten lncRNAs examined by more than 10-fold, suggesting possible roles of the prooxidants in the onset of some diseases. We will discuss the relevance of our findings below.

Recently, we identified two lncRNAs (Kcnq1ot1 and Rmst) that significantly decreased in mouse models of chronic liver diseases (such as MASH and fibrosis), and proposed that they serve as potential biomarkers for these diseases. These lncRNAs have also been proposed to be involved in the onset of several diseases: Kcnq1ot1 has been shown to promote cancer progression and metastasis [6,26,27]. Kcnq1ot1 also has been linked to oxidative stress-related diseases [28,29]. Rmst was implicated in several oxidative stress-related diseases such as cancer [30–32], neurological disorders [33] or atherosclerosis [34]. These findings clearly underscore the importance of these lncRNAs in certain diseases related with oxidative stress. Our findings show that 14 out of 20 prooxidants increased the expression level of Kcnq1ot1 and that 8 out of 20 increased that of Rmst. Quercetin, tyramine, *N*-methyl phenethylamine, 1,2,4-benzenetriol, 1,4-naphthoquinone, and oxyresveratrol, all of which increased both of these lncRNAs were reported to be prooxidants that generate ROS [21,24,25,35], suggesting the involvement of oxidative stress in the expression of the lncRNAs (Kcnq1ot1 and Rmst). Thus, our findings demonstrate a strong correlation between the expression of lncRNAs and chemical compounds capable of generating ROS. Further studies are required to clarify whether these compounds are involved in the pathogenesis of diseases by generating ROS and inducing the expression of these lncRNAs.

Next, we will discuss how twenty prooxidants with different structures differently induce the expression of lncRNAs *in vitro*. We first compare the effects of catecholamines and quinones. Among catecholamines, dopamine was reported to have the strongest ROS-generating activity (DNA-cleaving activity), followed by adrenaline and 6-hydroxydopamine *in vitro* [36]. Autooxidation of catecholamines is closely related to their ROS-generating activity [36]. These sequences appear inversely related to their induction effects on lncRNA expression levels examined in the present study: 6-hydroxydopamine, adrenaline, and dopamine induced the expression levels of 5, 4, and 1 lncRNA(s), respectively, by more than 5-fold. Dopamine with strong ROS-generating activity may be converted into other metabolites with weaker activity *in vitro*. There have been three main pathways of dopamine metabolism [37]. Dopamine is oxidatively deaminated to 3,4-dihydroxyphenylacetaldehyde (DOPAL) by monoamine oxidase (MAO) [37]. DOPAL was shown to be stronger oxidant than dopamine [38]. However, MAO is found in the central nerve system (CNS) or periphery [37], and thus, is not likely to be involved in the case of lncRNA generation in NIH3T3 cells, a fibroblast cell line. As another metabolic pathway, dopamine is converted to 3-methoxytyramine by catechol-*O*-methyltransferase (COMT), a ubiquitous enzyme that transfers methyl groups from *S*-adenosylmethionine (SAM) to hydroxyl groups of compounds with catechol moiety [39]. COMT has been reported to inactivate some compounds with catechol moiety and DNA-damaging activity [40]. In good agreement with this idea, 3-methoxytyramine was reported to have less cellular toxicity than dopamine, its non-methylated form [40]. COMT is ubiquitously localized, thus may explain the lowered ROS-generating activity or lncRNA-generating activity of dopamine metabolite. Alternatively, dopamine may undergo phase II metabolism and be conjugated with sulfate or glucuronic acid, by *O*-sulfatation or *O*-glucuronidation, respectively, prior to excretion [37]. ROS-generating activity of these possible dopamine metabolites described above may be closely related to their lncRNA-inducing activity.

Quinones (naphthoquinones and 1,2,4-benzenetriol) were found to efficiently increase the expression levels of all ten lncRNAs examined. 1,2,4-benzenetriol was reported to be a superior ROS generator compared to caffeic acid [41]. 1,2,4-benzenetriol was reported as a superior ROS-generating activity as well as toxicity [42]. 1,2-naphthoquinone was reported to have strong ROS (hydrogen peroxide or hydroxyl radical) -generating activity, followed by 1,4-naphthoquinone [25], thus demonstrating that 1,4-naphthoquinone is a relatively weaker ROS-generator compared with 1,2-naphthoquinone. However, we found in the present study that 1,4-naphthoquinone is a superior inducer of lncRNA expression when compared with 1,2-naphthoquinone. These results suggest that compounds with moderate rather than high ROS-generating ability may preferentially induce expression of target lncRNAs examined in this study. Although lines of evidences have shown that PM2.5 exerts its toxicological effects by regulating the expression of lncRNAs in several oxidative stress-related diseases [43–46], relatively little is known on the involvement of ROS and substances responsible for generation of ROS. Because 1,4-naphthoquinone is one of the known environmental pollutants contained in PM2.5 that can generate ROS, as discussed above, further studies are required to elucidate the possible toxic mechanism of the compound as well as those of 1,2,4-benzenetriol.

Lastly, we shall discuss why 1,4-naphthoquinone and 1,2,4-benzenetriol were superior lncRNA-inducers compared to other prooxidants such as flavonoids. Food-derived flavonoids or environmental pollutants (naphthoquinone) are often hydrophobic xenobiotics subject to modification mediated by phase II conjugation — that is, attachment of an ionized groups (including glutathione-, methyl- or acetyl-groups) to these compounds — thus making their metabolite more water-soluble [47,48]. UDP- glucuronosyltransferases (UGTs) catalyze glucuronidation of phenolics; thus altering their solubility, bioactivity, bioavailability, transport and excretion properties [49,50]. Glucuronidation of phenolics examined in this study (flavonoids such as fisetin, quercetin and chrysin; aromatic amines such as tyramine and dopamine) may have altered the ROS-generating or lncRNA-inducing activities as results of modification by metabolic enzymes. Methylation reactions on xenobiotics’ hydroxyl groups are catalyzed by methyl-*O*-transferases [51]. Catechol- *O*-methyltransferase (COMT) also catalyzes *O*-methylation on mutagenic flavonoids such as quercetin or fisetin; probably thereby suppressing their ROS-generating activities [49]. Zhu *et al.* reported flavonoids (quercetin or fisetin) as good substrates for COMT enzyme from porcine liver or hamster kidney [52]; whereas catecholamines (adrenaline or dopamine) were relatively poor substrates [52]. If a similar substrate specificity applies for COMT from NIH3T3 cells used here; flavonoids—but not catecholamines—would be preferentially metabolically-inactivated; leaving catecholamines as stronger ROS generators. This possibility must be examined in further studies, because, in good agreement with the assumption above, catecholamines (dopamine, 6-hydroxydopamine and adrenaline) were found as stronger lncRNA-inducer than flavonoids with catechol-moiety (luteolin, fisetin, quercetin) in the present study. To our knowledge, no reports exist regarding whether naphthoquinones, 1,2,4-benzenetriol or their metabolites act as COMT substrates. They may escape metabolic modifications by enzymes such as COMT or UGT; retaining their ROS-generating capacity along with strong lncRNA-inducing activities; whereas compounds undergoing metabolic modifications may lose these activities.

The present study is limited by lack of evidence explaining metabolism pathways for these prooxidants; mechanisms underlying their ROS-generation; and knowledge regarding how lncRNAs respond specifically under oxidative stress conditions *in vitro.* Further studies should focus on clarifying prooxidants’ precise roles via ROS generation pathways; alongside elucidation regarding potential roles played by identified lncRNAs within disease contexts. We believe our findings will contribute toward understanding both potential roles played by prooxidants as triggers for oxidative stress-related diseases, as well as functional implications held by specific lncRNAs within such disease states.

## Supporting information

S1 File RT-qPCR data tables. (XLSX)

## References

[1] Mattick JS, Amaral PP, Carninci P, Carpenter S, Chang HY, Chen L-L, et al. Long non-coding RNAs: definitions, functions, challenges and recommendations. Nat Rev Mol Cell Biol. 2023;24(6):430–47. doi: 10.1038/s41580-022-00566-8 36596869 PMC10213152

[2] Obuse C, Hirose T. Functional domains of nuclear long noncoding RNAs: Insights into gene regulation and intracellular architecture. Curr Opin Cell Biol. 2023;85:102250. doi: 10.1016/j.ceb.2023.102250 37806294

[3] Onoguchi-Mizutani R, Akimitsu N. Long noncoding RNA and phase separation in cellular stress response. J Biochem. 2022;171(3):269–76. doi: 10.1093/jb/mvab156 35080597

[4] Clark MB, Johnston RL, Inostroza-Ponta M, Fox AH, Fortini E, Moscato P, et al. Genome-wide analysis of long noncoding RNA stability. Genome Res. 2012;22(5):885–98. doi: 10.1101/gr.131037.111 22406755 PMC3337434

[5] Tani H, Mizutani R, Salam KA, Tano K, Ijiri K, Wakamatsu A, et al. Genome-wide determination of RNA stability reveals hundreds of short-lived noncoding transcripts in mammals. Genome Res. 2012;22(5):947–56. doi: 10.1101/gr.130559.111 22369889 PMC3337439

[6] Yokoyama S, Muto H, Honda T, Kurokawa Y, Ogawa H, Nakajima R, et al. Identification of Two Long Noncoding RNAs, Kcnq1ot1 and Rmst, as Biomarkers in Chronic Liver Diseases in Mice. Int J Mol Sci. 2024;25(16):8927. doi: 10.3390/ijms25168927 39201613 PMC11354866

[7] Kim C, Kang D, Lee EK, Lee J-S. Long Noncoding RNAs and RNA-Binding Proteins in Oxidative Stress, Cellular Senescence, and Age-Related Diseases. Oxid Med Cell Longev. 2017;2017:2062384. doi: 10.1155/2017/2062384 28811863 PMC5547732

[8] D’Autreaux B, Toledano MB. ROS as signalling molecules: mechanisms that generate specificity in ROS homeostasis. Nat Rev Mol Cell Biol. 2007;8:813–24. 17848967 10.1038/nrm2256

[9] Tani H, Numajiri A, Aoki M, Umemura T, Nakazato T. Short-lived long noncoding RNAs as surrogate indicators for chemical stress in HepG2 cells and their degradation by nuclear RNases. Sci Rep. 2019;9(1):20299. doi: 10.1038/s41598-019-56869-y 31889167 PMC6937343

[10] Tani H, Onuma Y, Ito Y, Torimura M. Long non-coding RNAs as surrogate indicators for chemical stress responses in human-induced pluripotent stem cells. PLoS One. 2014;9(8):e106282. doi: 10.1371/journal.pone.0106282 25171338 PMC4149554

[11] Hayakawa S, et al. Contribution of non-coding RNAs to anticancer effects of dietary polyphenols: Chlorogenic acid, curcumin, epigallocatechin-3-gallate, genistein, quercetin and resveratrol. Antioxidants. 2022;11:2352. 36552560 10.3390/antiox11122352 PMC9774417

[12] Jiang P, Chen A, Wu X, Zhou M, Ul Haq I, Mariyam Z, et al. NEAT1 acts as an inducer of cancer stem cell-like phenotypes in NSCLC by inhibiting EGCG-upregulated CTR1. J Cell Physiol. 2018;233(6):4852–63. doi: 10.1002/jcp.26288 29152741

[13] Geng W, Guo X, Zhang L, Ma Y, Wang L, Liu Z, et al. Resveratrol inhibits proliferation, migration and invasion of multiple myeloma cells via NEAT1-mediated Wnt/β-catenin signaling pathway. Biomed Pharmacother. 2018;107:484–94. doi: 10.1016/j.biopha.2018.08.003 30107344

[14] Sheng B, Zhao L, Zang X, Zhen J, Liu Y, Bian W, et al. Quercetin inhibits caerulein-induced acute pancreatitis through regulating miR-216b by targeting MAP2K6 and NEAT1. Inflammopharmacology. 2021;29(2):549–59. doi: 10.1007/s10787-020-00767-7 33051781

[15] Lee KW, Lee HJ. The roles of polyphenols in cancer chemoprevention. Biofactors. 2006;26(2):105–21. doi: 10.1002/biof.5520260202 16823097

[16] Halliwell B, Gutteridge JM. Role of free radicals and catalytic metal ions in human disease: an overview. Methods Enzymol. 1990;186:1–85. doi: 10.1016/0076-6879(90)86093-b 2172697

[17] Sakihama Y, Cohen MF, Grace SC, Yamasaki H. Plant phenolic antioxidant and prooxidant activities: phenolics-induced oxidative damage mediated by metals in plants. Toxicology. 2002;177(1):67–80. doi: 10.1016/s0300-483x(02)00196-8 12126796

[18] Procházková D, Boušová I, Wilhelmová N. Antioxidant and prooxidant properties of flavonoids. Fitoterapia. 2011;82(4):513–23. doi: 10.1016/j.fitote.2011.01.018 21277359

[19] Li Z, Yang X, Dong S, Li X. DNA breakage induced by piceatannol and copper(II): Mechanism and anticancer properties. Oncol Lett. 2012;3(5):1087–94. doi: 10.3892/ol.2012.597 22783397 PMC3389628

[20] Borah A, Paul R, Mazumder MK, Bhattacharjee N. Contribution of β-phenethylamine, a component of chocolate and wine, to dopaminergic neurodegeneration: implications for the pathogenesis of Parkinson’s disease. Neurosci Bull. 2013;29(5):655–60. doi: 10.1007/s12264-013-1330-2 23575894 PMC5561952

[21] Kawano T. Prion-derived copper-binding peptide fragments catalyze the generation of superoxide anion in the presence of aromatic monoamines. Int J Biol Sci. 2006;3:57–63. 17200692 10.7150/ijbs.3.57 PMC1657085

[22] Rehmani N, Zafar A, Arif H, Hadi SM, Wani AA. Copper-mediated DNA damage by the neurotransmitter dopamine and L-DOPA: A pro-oxidant mechanism. Toxicol In Vitro. 2017;40:336–46. doi: 10.1016/j.tiv.2017.01.020 28137434 PMC5404347

[23] Goldstein LE, Leopold MC, Huang X, Atwood CS, Saunders AJ, Hartshorn M, et al. 3-Hydroxykynurenine and 3-hydroxyanthranilic acid generate hydrogen peroxide and promote alpha-crystallin cross-linking by metal ion reduction. Biochemistry. 2000;39(24):7266–75. doi: 10.1021/bi992997s 10852726

[24] Hiramoto K, Li X, Makimoto M, Kato T, Kikugawa K. Identification of hydroxyhydroquinone in coffee as a generator of reactive oxygen species that break DNA single strands. Mutat Res. 1998;419(1–3):43–51. doi: 10.1016/s1383-5718(98)00123-5 9804887

[25] Charrier JG, Anastasio C. Rates of Hydroxyl Radical Production from Transition Metals and Quinones in a Surrogate Lung Fluid. Environ Sci Technol. 2015;49(15):9317–25. doi: 10.1021/acs.est.5b01606 26153923 PMC4777526

[26] Liu G, Shi L, Wang B, Wu Z, Zhao H, Zhao T, et al. Role of oncogenic long noncoding RNA KCNQ1OT1 in colon cancer. Oncol Res. 2024;32:585–96. 38361755 10.32604/or.2023.029349 PMC10865742

[27] Cagle P, Qi Q, Niture S, Kumar D. KCNQ1OT1: An Oncogenic Long Noncoding RNA. Biomolecules. 2021;11(1602). 10.3390/biom11111602 PMC8615887 34827600

[28] Yang F, Qin Y, Lv J, Wang Y, Che H, Chen X, et al. Silencing long non-coding RNA Kcnq1ot1 alleviates pyroptosis and fibrosis in diabetic cardiomyopathy. Cell Death Dis. 2018;9(10):1000. doi: 10.1038/s41419-018-1029-4 30250027 PMC6155223

[29] Xu C, Chen L, Wang R-J, Meng J. LncRNA KCNQ1OT1 knockdown inhibits ox-LDL-induced inflammatory response and oxidative stress in THP-1 macrophages through the miR-137/TNFAIP1 axis. Cytokine. 2022;155:155912. doi: 10.1016/j.cyto.2022.155912 35598525

[30] Cai H, Li C, Wu Z. lncRNA RMST is associated with the progression and prognosis of gastric cancer via miR-204-5p. Cell Div. 2024;19(1):12. doi: 10.1186/s13008-024-00117-x 38610003 PMC11015603

[31] Chen S, Ji L, Wang Y, Zhang L, Xu M, Su Y, et al. lncRNA RMST suppresses the progression of colorectal cancer by competitively binding to miR-27a-3p/RXRα axis and inactivating Wnt signaling pathway. Acta Biochim Biophys Sin (Shanghai). 2023;55(5):726–35. doi: 10.3724/abbs.2023065 37246895 PMC10281887

[32] Tani H. RMST: a long noncoding RNA involved in cancer and disease. J Biochem. 2025;177(2):73–8. doi: 10.1093/jb/mvae083 39673327

[33] Zhou L, Zhi Z, Chen P, Du C, Wang B, Fang X, et al. LncRNA-RMST Functions as a Transcriptional Co-regulator of SOX2 to Regulate miR-1251 in the Progression of Hirschsprung’s Disease. Front Pediatr. 2022;10:749107. doi: 10.3389/fped.2022.749107 35321017 PMC8936393

[34] Zhang T, Feng C, Zhang X, Sun B, Bian Y. Abnormal expression of long non-coding RNA rhabdomyosarcoma 2-associated transcript (RMST) participates in the pathological mechanism of atherosclerosis by regulating miR-224-3p. Bioengineered. 2022;13(2):2648–57. doi: 10.1080/21655979.2021.2023995 35067166 PMC8974166

[35] Yamashita N, Tanemura H, Kawanishi S. Mechanism of oxidative DNA damage induced by quercetin in the presence of Cu(II). Mutat Res. 1999;425(1):107–15. doi: 10.1016/s0027-5107(99)00029-9 10082921

[36] Spencer WA, Jeyabalan J, Kichambre S, Gupta RC. Oxidatively generated DNA damage after Cu(II) catalysis of dopamine and related catecholamine neurotransmitters and neurotoxins: Role of reactive oxygen species. Free Radic Biol Med. 2011;50(1):139–47. doi: 10.1016/j.freeradbiomed.2010.10.693 21075203 PMC3353411

[37] Meiser J, Weindl D, Hiller K. Complexity of dopamine metabolism. Cell Commun Signal. 2013;11(1):34. doi: 10.1186/1478-811X-11-34 23683503 PMC3693914

[38] Li SW. 3,4-dihydroxylphenylacetaldehyde and hydrogen peroxide generate a hydroxyl radical: a possible role in Parkinson’s disease pathogenesis. Brain Research: Molecular Brain Research. 2001;93:1–7. 11532332 10.1016/s0169-328x(01)00120-6

[39] Xie T, Ho SL, Ramsden D. Characterization and implications of estrogenic down-regulation of human catechol-O-methyltransferase gene transcription. Mol Pharmacol. 1999;56:31–8. 10385681 10.1124/mol.56.1.31

[40] Blessing H, Bareiss M, Zettlmeisl H, Schwarz J, Storch A. Catechol-O-methyltransferase inhibition protects against 3,4-dihydroxyphenylalanine (DOPA) toxicity in primary mesencephalic cultures: new insights into levodopa toxicity. Neurochem Int. 2003;42(2):139–51. doi: 10.1016/s0197-0186(02)00075-x 12421594

[41] Li Y, Trush MA. Reactive oxygen-dependent DNA damage resulting from the oxidation of phenolic compounds by a copper-redox cycle mechanism. Cancer Res. 1994;54(7 Suppl):1895s–8s. 8137307

[42] Singh V, Ahmad S, Rao GS. Prooxidant and antioxidant properties of iron-hydroquinone and iron-1,2,4-benzenetriol complex. Implications for benzene toxicity. Toxicology. 1994;89:25–33. 7513907 10.1016/0300-483x(94)90130-9

[43] Huang Q, et al. Fine particulate matter 2.5 exerted its toxicological effect by regulating a new layer, long non-coding RNA. Sci Rep. 2017;7:9392. 28839203 10.1038/s41598-017-09818-6 PMC5570922

[44] Li X, Zheng M, Pu J, Zhou Y, Hong W, Fu X, et al. Identification of abnormally expressed lncRNAs induced by PM2.5 in human bronchial epithelial cells. Biosci Rep. 2018;38(5):BSR20171577. doi: 10.1042/BSR20171577 29899163 PMC6131355

[45] Aghaei-Zarch SM, Alipourfard I, Rasoulzadeh H, Najafi S, Aghaei-Zarch F, Partov S, et al. Non-coding RNAs: An emerging player in particulate matter 2.5-mediated toxicity. Int J Biol Macromol. 2023;235:123790. doi: 10.1016/j.ijbiomac.2023.123790 36822288

[46] Tian P, Xia H, Li X, Wang Y, Hu B, Yang Y, et al. Identification and Assessment of lncRNAs and mRNAs in PM2.5-Induced Hepatic Steatosis. Int J Mol Sci. 2025;26(6):2808. doi: 10.3390/ijms26062808 40141450 PMC11943408

[47] Almazroo OA, Miah MK, Venkataramanan R. Drug Metabolism in the Liver. Clin Liver Dis. 2017;21:1–20. 27842765 10.1016/j.cld.2016.08.001

[48] Jancova P, Anzenbacher P, Anzenbacherova E. Phase II drug metabolizing enzymes. Biomed Pap Med Fac Univ Palacky Olomouc Czech Repub. 2010;154(2):103–16. doi: 10.5507/bp.2010.017 20668491

[49] Turgeon D, Carrier J-S, Chouinard S, Bélanger A. Glucuronidation activity of the UGT2B17 enzyme toward xenobiotics. Drug Metab Dispos. 2003;31(5):670–6. doi: 10.1124/dmd.31.5.670 12695357

[50] Wu B, Basu S, Meng S, Wang X, Hu M. Regioselective sulfation and glucuronidation of phenolics: insights into the structural basis. Curr Drug Metab. 2011;12(9):900–16. doi: 10.2174/138920011797470100 21933112 PMC3426368

[51] Chen Z, Zheng S, Li L, Jiang H. Metabolism of flavonoids in human: a comprehensive review. Curr Drug Metab. 2014;15(1):48–61. doi: 10.2174/138920021501140218125020 24588554

[52] Zhu BT, Ezell EL, Liehr JG. Catechol-O-methyltransferase-catalyzed rapid O-methylation of mutagenic flavonoids. Metabolic inactivation as a possible reason for their lack of carcinogenicity in vivo. J Biol Chem. 1994;269(1):292–9. 8276810

